# A Systematic Review and Metasynthesis of Hematopoietic Stem Cell Transplant (HSCT) Patient's Experiences of Long-Term Monitoring Clinics from the Patient's Perspective

**DOI:** 10.1177/23743735241229378

**Published:** 2024-02-08

**Authors:** Blossom Bell, Stacie Thursby, Helen Limbrick, Katherine Swainston

**Affiliations:** 1 98273 Population Health Sciences Institute, Newcastle University, Newcastle upon Tyne, UK; 2 Northumbria Healthcare NHS Foundation Trust, North Tyneside General Hospital, Rake Lane, Tyne and Wear, North Shields, UK; 3 School of Social Sciences, Humanities & Law, 5462 Teesside University, Middlesbrough, Tees Valley, UK; 4 School of Psychology, 12186 Faculty of Medical Sciences, Newcastle University, Newcastle upon Tyne, UK

**Keywords:** stem cell transplant, patients’ experiences, long-term monitoring, cancer recurrence

## Abstract

This study aimed to synthesize all qualitative evidence on the experiences of hematopoietic stem cell transplant (HSCT) patients attending long-term monitoring clinics from their perspective. A systematic search of the literature was undertaken across 8 databases. The Critical Appraisal Skills Program was used to evaluate each study's quality. Confidence in the Evidence from Reviews of Qualitative Research was employed to assess confidence in each finding. Three themes from 4 qualitative studies were identified relating to patients’ experiences, “[It's] important to maintain a good relationship with the nurses and doctors,” “There's always the thing about the logistics,” and “Once you have cancer, you’re always thinking do I have it again?”. The findings suggest that HSCT patients’ experiences of long-term follow-up care clinics are influenced by the patient-provider relationship and the logistical set-up of monitoring practices, and weakly connected with fear of cancer recurrence. Future research is needed to understand the impact of each finding of this review, specifically in relation to patients’ country of residence to gain a greater understanding of their monitoring support needs.

## Introduction

A hematopoietic stem cell transplant (HSCT) is a potentially curative treatment option for malignant hematological disorders. It is a procedure that replaces defective stem cells with new healthy ones, mainly sourced from either a donor (allogeneic) or the patient (autologous), to restore lost hematopoietic function.^1^ It was estimated that more than 1.5 million HSCTs were performed worldwide between 1957 and 2019, and annual rates continue to increase without plateau.^2^ In 2019, Europe (and collaborating countries) reported 48,512 HSCTs (41% allogeneic and 59% autologous) performed, a 2.2% increase compared to 2018 (n = 47,468).^3^ The continuing annual increase of HSCTs performed, combined with improved survival rates within the first year of transplant has substantially increased the number of HSCT survivors. In the United States alone, it is estimated that there will be just over half a million HSCT survivors by 2030.^4^ HSCT survivorship does not mark the end of the care trajectory for HSCT patients due to the increased risk of treatment-related mortality.

While advances in medical treatments and patient management have contributed to reducing mortality rates for HSCTs patients, rates remain higher than the general population. Treatment-related complications include infections, relapse, graft-versus-host disease (GVHD), subsequent cancer, and organ dysfunction. Relapse is frequently reported in studies as being the leading cause of mortality. Nonrelapse mortality was primarily related to post-transplantation infections and secondary cancers.^5–7^ In recognition of these late effects and the impact on mortality, transplant centers (TCs) must provide long-term follow-up (LTFU) for post-HSCT patients.^8^ Accreditation standards stipulate that TCs are required to monitor recipients’ endocrine, reproductive, cardiovascular, renal, and respiratory function, and for osteoporosis and secondary cancer risks. Monitoring should be carried out by dedicated clinics structured in line with international guidelines and literature, which includes recommended screening practices and test frequencies. The guidelines also recommend that LTFU clinics encourage health promotion behaviors such as maintaining a health diet and weight, refrain from smoking, moderate alcohol use, and avoiding excessive sun exposure.^9^

Despite the international guidelines and accreditation standards, the continuation of post-transplant LTFU by TC is known to be variable. Studies examining the implementation of such guidelines and literature from TC's perspective found variations in service, including fragmented care, access to cancer screening and inclusion of psychological support. Funding, patient demographics, and lack of capacity and physicians were the reasons behind monitoring disparities.^10–12^ In recognition of a growing population of HSCT survivors, transplant-related mortality risk and known variation in TC care, the focus has shifted to the management and healthcare infrastructure of long-term monitoring to deliver a patient-centered service.^13,14^ If TCs are going to provide a patient-centered service then understanding HSCT patients’ experiences of attending long-term monitoring appointments is crucial to achieving this objective. Despite this, to date, a systematic review of qualitative studies has not been conducted, and the literature lacks an overview of findings on patients’ experiences.

This systematic review aims to appraise and synthesize the qualitative evidence of HSCT patients’ experiences of long-term monitoring clinics to ascertain what is currently known, identify gaps in the literature, and make recommendations for future research. The review protocol is registered with PROSPERO [Registration number CRD42021231860).

## Method

**Figure 1.**
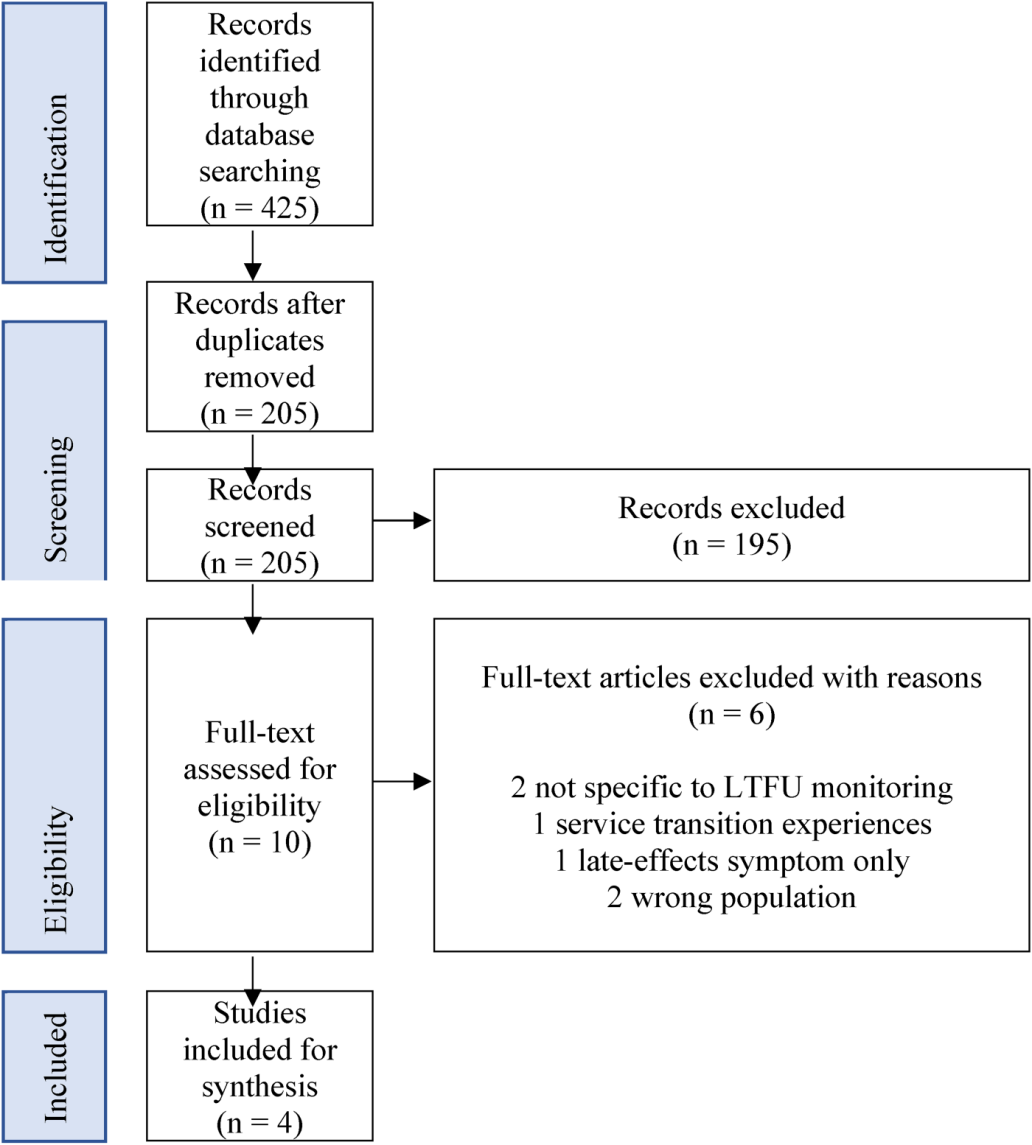
PRISMA results.

A systematic search was undertaken using the population, context, outcome framework to identify keywords in the review question. Once the keywords were identified a list of all synonyms were developed to form the basis of the search strategy. Eight databases were searched (Psych Info, Medline, The Allied and Complementary Medicine Database, CINAHL, Psychology and Behavioral Science Collection, Scopus, Embase, and Proquest Nursing and Allied Health Source). Year parameter was set for articles published from January 2006 to December 2022, in line with the first published recommended screening and preventive practice for HSCT survivors.^15^ The initial search retrieved 425 references. After the removal of duplicates, the title and abstract of the remaining 203 papers were screened blindly by 2 reviewers (the first and last authors) in software for systematic reviews, Rayyan^©^ (www.rayyan.ai). Reviewers used the following inclusion criteria: (i) patients received a stem cell transplant + 100 days prior to the study, (ii) qualitative studies to include phenomenology, ethnography, grounded theory, generic qualitative or mixed methods which examined patients’ experiences specific to long-term monitoring services, (iii) English language. Ten papers were selected for full-text review by the first and second authors and any disagreements were resolved in discussion with the third author. Papers were excluded if they (i) did not elicit responses directly from patients and (ii) focused on monitoring late-effects symptoms only or (iii) experiences not related to LTFU care. In total 6 articles were removed (Figure 1).

Four studies were included for the final synthesis (Table 1). Seventy HSCT patients were included in the 4 studies, 3 of which included only patients who received an allo-HSCT, with only 1 study including one allo-HSCT patient.^16–19^ Mean time since transplant across the studies ranged from 2.7 to 8.13 years (range 0.25-20 years). The studies were undertaken in the UK, USA, Germany, and Singapore. One study recruited participants from a designated LTFU clinic, 2 recruited from a clinic dedicated to GVHD, and 1 recruited participants from their hospital registry. The Critical Appraisal Skills Program (CASP) tool for qualitative research was used for quality appraisal by the first and second authors. The guiding methodology for this study was thematic synthesis, as outlined by Thomas and Harden, to elicit a rigorous high-level analytical abstraction of common themes across studies.^20^ The eligible studies were inputted verbatim into MAXQDA 2022 software for storing, coding, and data search. All text labeled as “results” or “findings” in each study were examined line-by-line for meaning and content by the first and second authors and free codes were generated. The free codes generated that reflected the patients experiences of attending LTFU clinics were then grouped into related areas to construct descriptive themes which captured the meaning of groups of the initial free codes. The descriptive themes were then synthesized, and analytical themes were developed to generate additional concepts and understanding of patients experiences beyond the findings of the primary studies. To ensure that all patients’ experiences had been captured, the studies were read several times and discussed at length between the first and second author. Any interpretive disagreements were discussed with and resolved by the third author. The Confidence in the Evidence from Reviews of Qualitative (GRADE-CERQual) research was used to determine the overall confidence in the study findings.^21^ An estimation of moderate and high confidence for the findings was reached (Table 2).

**Table 1.** Summary of Included Studies.

Study No.	Authors/Year/Country	Population	Aim	Method	Themes identified by primary study authors	CASP (10)
1	Hwang et al,(2012). USA	22 allo-HSCT patients (11 males and 11 females). Age range 22-69 years. Mean time since transplant was 5.2 years (range 2.4-8 years).	To explore the attitudes of long-term survivors of HSCT about their healthcare use and information needs	Thematic analysis using grounded theory approach. Three focus groups and 12 structured interviews conducted.	Late effects; healthcare issues; health education and outreach; personal factors; social factors	10
2	Parisek et al, (2021). Germany	32 allo-HSCT survivors (16 males and 16 females) and 18 partners. Age range of survivors was 25-68 years. Mean time since transplant was 2.7 years (range 1.2 - 5 years). Attended GVHD clinic for routine follow-up or second opinions.	To explore in-depth HSCT survivors’ experiences and needs post-transplant. Partners included to provide further information.	Framework method of analysis of semistructured telephone interviews.	Diversity of long-term treatment side-effects as a major challenge in alloHSCT survivorship care; Time post-discharge as a dynamic process with individual peaks of burden; Transparent communication and support with patient empowerment; Continuity in the care system and help with claiming social benefits as cornerstones of optimal survivorship care.	10
3	Sharin et al, (2021). Singapore.	8 HSCT patients (1 auto & 7 allo-HSCT) (3 males and 5 females). Age range 27-67 years. Mean time since transplant was 8.13 years (range 4-20 years). At least one visit to long-term follow-up clinic.	To explore the experiences of HSCT survivors attending a long-term follow-up clinic	Qualitative thematic analysis of one-to-one semistructured interviews.	Comprehending the experiences; acknowledging the meaningfulness of the experience; managing threats to a new life after transplant	10
4	de Vere Hunt et al, (2021). UK	8 allo-HSCT patients (2 males and 2 females). Age range of survivors 46-68 years. Mean time since transplant 3 years (range 0.25-15 years). Attended GVHD clinic.	Exploration of patient perspectives of their HRQOL and experiences of service provision in a multidisciplinary specialist care clinic.	Qualitative thematic analysis of one-to-one semistructured interviews.	Themes related to QOL issues in GVHD; *‘*Restricted as to what I can do’; Troubling symptoms—“*you can sort of get GVHD anywhere”*; Confusion and uncertainty regarding GVHD symptoms—“*Is this the GVHD?”*; Unpredictable course and uncertainty regarding the future; Adapting to the sick role Themes related to patient experience of the healthcare service provision; Personal care and close relationship with BMT nurses; Efficiency versus long waiting times—“on the case straight away”; Information provision—“*went into it with a bit of a rosy view”*; The role of support groups	10

CASP, Critical Appraisal Skills Program; GVHD, graft-versus-host disease; HRQOL, health-related QOL.

**Table 2.** CERQual Summary of Qualitative Findings Table.

Objective: To explore and synthesize the qualitative evidence pertaining to HSCT patients’ experiences of long-term monitoring clinics and associated impact on FCR				
Perspective: Experiences of HSCT patients’ attending long-term monitoring clinics and associated impact on FCR				
Summary of review findings	Supporting quotes (with study number)	Studies contributing to the review findings	*CERQual assessment of confidence in the evidence*	Explanation of CERQual assessment
“Important to maintain good relationship with the nurses and doctors”: Patients establishing relationships with clinical personnel was seen as a crucial element to receiving personal and attentive care, as well as maintaining health	*You know, as far as sort of care and attention is concerned from the people … I can’t fault it really.”* (4) *you see … how important to maintain good relationship with others who cares for you even the nurses and doctors* (1) *I’ve had ready access to XXX, the post-transplant nurse co-ordinator, and she's been very good; seems very knowledgeable and very encouraging… so I think a personal relationship is important. And the fact that she has access to all of the other colleagues that she can then go and speak to and get back to me.* (4) *I really have to praise it—this wonderful system of building a bridge between hospital care and care at home. There is someone who takes the time to tell us what to look out for at home.* (2) “*Every time I build a relationship with a doctor they leave soon after. It is really annoying. Because you always talk a bit about yourself. First, the doctor is a stranger to you, then, during the second visit, you start to get to know them, and again during the third visit, and when you want to meet them for the fourth time they are gone.”* (2) “*I feel very comfortable [with the cancer specialists]—like they really do care about you. [O]ne year I got real sick, and I ended up going to a [noncancer specialist]. And they made me feel like they weren’t solving the problem”* (1) *in terms of the expertise and the consultants I see, I’ve got no real issue. It's just that I see lots of different people rather than the same person* (4)	1, 2, 3, 4	High confidence	Four studies with no or very minor concerns about coherence, relevance, adequacy, and methodological limitations.
*“There's always the thing about the logistics”*: Patients identified several logistical problems with attending monitoring clinic appointments, such as, delays, length, frequency, and financial consequences.	*“I’ve been impressed basically in terms of, you know, efficiency… it all seems to be, you know, well organized, efficient, or reasonably efficient when I go, you know, you’re never seen exactly on time but, you know, you do get seen… people do spend time with you… from my point of view it seems to work quite well; very well, not just quite well; very well”* (4) ‘*Occasionally, it doesn’t happen very often now, but there are occasions where it is a long wait… But then I know that when I was poorly I had the attention of my consultant… there was no rush if you know what I mean, so I appreciate that there would be someone somewhere in with somebody who's very poorly and is taking a long time more, which I understand’* (4) ‘*I see my [eye doctor and a doctor for] GVHD… [Appointments] pile up during the day…I’m here from 8:00 in the morning until 4:00 in the afternoon… [Y]ou just get tired of waiting and sitting’* (1) “*All these appointments are not cheap. It is very expensive. If having more appointments as the days goes by, it means the expenses will be more for us. Sometimes I even ask them whether it is important for these appointments. I understand all these, I need to spend”* (3) “*The once that I asked to see a dermatologist I was able to see her that same day, so that was reassuring”* (4) *Participants reported a lack of communication between their treating specialists, GPs and the emergency departments they attended. GPs and local emergency departments were often not aware of the special needs of allo HSCT-survivors* (2) “*I’ll call cancer care and I’ll Google the cancer patient resources. There is help, but only for people in treatment. It's like they’ve forgotten the people after they’ve survived*” (1)	1, 2, 4	High confidence	Four studies with no or very minor concerns about coherence, relevance, adequacy, and methodological limitations.
***“*** *Once you have cancer, you’re always thinking do I have it again?**”***: Patients reported a fear of recurrence and uncertainty of the future, feelings of which were associated with monitoring practice.	*‘I feel very covered. I feel very safe in [how much] screening they do’* (1) ‘*If you did less [screening], you’ll always have that in your head, ‘[Do] I have something?’ Once you have cancer you’re always thinking …do I have it again’* (1) “*The ultrasound image showed up something on my thyroid. And then they [ = the treating clinician] say to you: ‘Well, we’ll definitely have to investigate that. You were getting radiation therapy and some tumors can come back.’ And my reaction was: danger, danger, danger!”* (2)	1, 2, 3	Moderate confidence	Two studies with no or very minor concerns about coherence, relevance, adequacy, and methodological limitations. One study with no or very minor concerns about, relevance, adequacy, and methodological limitations but a minor concern with coherence.

Abbreviations: CERQual, confidence in the evidence from reviews of qualitative; HSCT, hematopoietic stem cell transplant; FCR, fear of cancer recurrence; GVHD, graft-versus-host disease; GP, general practitioners.

## Results

The synthesis of results identified 3 analytical themes regarding recipients’ experiences of monitoring clinics. These were (i) “*[It's] important to maintain a good relationship with the nurses and doctors”*, (ii) “*There's always the thing about the logistics”*, and (iii) “*Once you have cancer, you’re always thinking do I have it again?”*.

“*[It's] important to maintain a good relationship with the nurses and doctors”*: Patients establishing relationships with clinical personnel was seen as a crucial element to receiving personal and attentive care, as well as maintaining health. Speaking to staff who already knew their medical history was both comforting and increased efficiency for patients. Furthermore, patients trusted personnel directives and valued them as a source of information.

While there was a general validation of the importance of a patient/provider relationship, patients frequently highlighted a distinction between the nurse-patient and doctor-patient relationships. Establishing close relationships with clinical liaison nurses was associated with maintaining clinical support. Nurses are seen as the primary facilitator of patient care and the main communicator among healthcare professionals. In contrast, establishing close relationships with clinical doctors was correlated with building patients’ confidence and becoming proactive in their care and a source of comfort. However, establishing such a relationship was hindered by patients not seeing the same clinician each time. Furthermore, if physicians were not specialists, many patients felt that they were not as informed or helpful in addressing their needs.

“*There's always the thing about the logistics”*: While some patients reported being satisfied with the organization and structure of clinics, others identified several logistical problems. In 1 study, several patients experienced long waiting periods, either at clinical appointments or with associated services such as hematology and pharmacy; however, sympathy for busy staff and the urgent needs of others mitigated levels of frustration caused. Other patients reported struggling with the length and frequency of appointments, usually requiring patients to commit to a full day, which meant prolonged times away from employment and preventing patients from making life-meaning plans. The frequency of appointments also had a financial implication for patients as attending was costly, causing some patients to question if some appointments were even needed. However, maintaining their health led patients to accept the financial implications as part of the care trajectory.

Despite the delays and organizational issues, patients did report clinics responding quickly to their concerns, especially if worries were regarding GVHD and clinics were formulated as a multidisciplinary team. The quick referrals and appointment access streamlined the process and provided reassurance. However, patients reported communication issues between monitoring clinics and other medical departments, including general practitioners (GPs) and accident and emergency care (A&E). Patients perceived other departments to lack the medical knowledge and experience of the long-term consequences of HSCT and that this barrier prevented their needs from being met or intensified feelings of abandonment.

“*Once you have cancer, you’re always thinking do I have it again?”*. Due to concerns regarding late complications and fear of recurrence, patients expressed the need to control their health which caused them to become more observant of the importance of long-term monitoring, screening practices, and tests. Concerning screening participation, some patients reported being satisfied and reassured with the level of screening undertaken. However, for other patients, the fear of recurrence fueled them to believe that they were not participating enough. How physicians reported the findings of screening tests was also anxiety-provoking, especially concerning physicians thinking out loud or not communicating in nonspecialists’ terms, consequently leading patients to fear that something was wrong and causing them distress.

## Discussion

This qualitative metasynthesis provides the first known systematic review of qualitative studies exploring the experiences of HSCT patients attending long-term monitoring clinics. This review revealed that the relationship status with healthcare professionals and logistical arrangements impacted HSCTs’ experiences in long-term monitoring clinics. Furthermore, attending appointments and participating in screening activities was reassuring for some but problematic for others due to concerns, worry or fear of cancer recurrence (FCR).^22^

Patients perceived liaison nurses as the primary facilitator of patient care and the main communicator among healthcare professionals, and establishing close relationships was essential to maintaining clinical support. However, this perceived reliance on liaison nurses may be influenced by the clinical management structure rather than a relationship status. Out of 19 TCs in the United Kingdom, ten were physician-led, 3 nurse-led, and 6 combined.^10^ Furthermore, physician availability may influence the dependence on liaison nurses as it has been reported that physicians within monitoring clinics are allocated less than one-third of their time (31.7%) on HSCT-related tasks, with much of their time being spent in general hematology.^23^ Nevertheless, the findings of this study suggest that patients view clinics as their primary care providers, which may become problematic given the expected number of survivors over the next decade and if TCs monitor follow-up via primary healthcare providers. Therefore, this represents an opportunity for increased research focused on understanding patients’ and providers’ expectations and roles within the 2 main monitoring processes stated in the TC accreditation manual.

Accreditation standards state that TCs perform long-term monitoring directly or coordinate care directly with referring physicians to prevent patients from being lost for follow-up.^9^ TCs choosing the latter may be hampered by the lack of communication patients reported between clinics, GPs, and A&E departments. The communication barrier prevented patients’ needs from being met or intensified feelings of abandonment. Consistent with other work, poor communication in healthcare can lead to various adverse outcomes, including patient dissatisfaction and discontinuity of care.^24^ However, each primary study originated from different countries with varying healthcare systems. Researching patients’ experiences within the same demographic location will provide a greater understanding of any communication barriers specific to the national healthcare structure to ensure patients’ needs are being met or identify areas for improvement. This will also enable the assessment of variation in patients’ experiences monitored via primary healthcare providers compared with patients being monitored directly by TCs.

Another notable finding was the variability in how monitoring practices impact FCR, especially concerning the number of screening tests undertaken. Some patients felt reassured by the amount of screening undertaken; for others, their FCR led them to believe that they were not attending enough. FCR was also reported to have been impacted by physicians’ consultations following the test. While the findings in this study were heterogeneous; they are comparable to survivors of breast cancer who perceived attending monitoring clinics as contradictory; attendance was reassuring while also exacerbating their FCR.^25^ Given the increased risk of treatment-related mortality and HSCT patients’ heightened awareness of late complications, attentional bias could explain the impact on patients’ FCR.^26,27^ Patients who report being more observant of screening practices has been found to be positively associated with increased levels of FCR.^28^ However, in a study of 335 allogeneic transplant patients no mean differences in FCR were found in patients who did or did not attend recommended screening despite 95% of survivors reported living with moderate (84%) to severe (11%) FCR. The only significant difference in FCR score was found in female participants who had not attended a cervical smear post-transplant.^29^ The relationship between FCR and LTFU screening practices is complex and given the paucity of research in this area,^30^ it is unclear if screening attendance or avoidance causes FCR or results from it. While the findings of this review suggest a weak connection between LTFU monitoring and FCR further research is required to understand the relationship in its entirety.

Regardless of the underlying causation, FCR heightens the importance of LTFU attendance for patients in this review as well as influences the need to control their health. Participating in health-promoting behaviors such as maintaining a healthy diet and weight, refrain from smoking, moderate alcohol use, and avoiding excessive sun exposure are all in patients’ control. However, a study of 664 HSCT survivors found that despite increased health monitoring, there was no significant improvement in health behaviors after treatment.^31^ In addition, HSCT survivors were less likely to participate in positive health behaviors such as physical activity,^32^ and cancer survivors who participated in negative health behaviors were more likely to experience FCR.^33^ Effectively managing patients’ FCR was associated with improving survivors’ quality of life and recommendations for FCR management included educational and support programs both prior to and post HSCT and screening for FCR at LTFU appointments.^29^ Yet, studies into TCs monitoring procedures have reported insufficient access to psychological support; therefore, patients may not have received the appropriate support to mitigate FCR impact.^10–12^ While the findings of this review highlight patients need to control health, what this actually means and its relations with FCR and the educational support available is unclear from our findings and further research is recommended.

As the first review exploring the experiences of HSCT patients attending long-term monitoring clinics this study has several strengths, including moderate/high confidence in the findings and a reproducible search strategy. Nonetheless, the findings are limited due to the paucity of research in this area, and that only 4 primary studies were identified that included HSCT patients experiences of attending LTFU clinics. Furthermore, the primary studies included in this review were from different countries with different healthcare structures; therefore, the findings may not be a true reflection of patients’ experiences within specific healthcare settings. For example, patients’ experiences of screening practice and FCR were drawn from 3 studies and included patients in the US, Singapore, and Germany. Therefore, for HSCT patients based in other countries, the experiences may be different. Take the UK for example, cervical and mammogram screening for HSCT patients relies on national screening programs, which access to is stated as problematic.^10^ It is therefore recommended that more research into patients experiences of LTFU care conducted in different countries to gain a greater understanding of HSCT patients experiences within specific health care structures.

Secondly, although studies included a high-quality score, 2 papers recruited participants from a clinic dedicated to monitoring GVHD; therefore, patients were under active care, which may have resulted in more attendance requirements resulting in a heightened awareness of logistical issues. It is therefore recommended that future research should focus on patients in LFTU but not under active care to get a fuller understanding of this cohort of patients’ experiences. In addition, 1 paper was published in 2012 and while TCs had a responsibility to monitor patients post-transplant it was not made mandatory until 2015.^8^ Therefore, the patients experiences within this paper may not reflect changes in LTFU practices since 2015. Finally, this review included studies with patients at 100+ days from transplant; however, patients may not have been formally discharged to long-term monitoring services at this stage. It is difficult to distinguish between post-HSCT monitoring and long-term monitoring as TCs sometimes incorporate both services into the same clinic.^12^

## Conclusion

The findings suggest that HSCT patients’ experiences of LTFU care clinics are influenced by the patient-provider relationship and the logistical set-up of monitoring practices. Opportunities exist to extend future research to understand the impact of each finding further and should focus on 2 key areas; examine patient and providers’ expectations and roles of monitoring services and cross-departmental communication specific to patients’ demographical location. Further understanding of roles, responsibilities and communication needs may reduce patient reliance on LTFU clinics for general healthcare needs, promote self-management where applicable, and identify barriers and facilitators to find potential solutions. In addition, the findings suggest a connection between monitoring clinics and patients’ FCR, however further research, including the potential relationship with associated health behaviors, is required to understand the relationship entirely and to gain a greater understanding of HSCT patients’ support needs.
